# Can we prevent childhood Leukaemia?

**DOI:** 10.1038/s41375-021-01211-7

**Published:** 2021-04-09

**Authors:** Mel Greaves, Valeria Cazzaniga, Anthony Ford

**Affiliations:** Centre for Evolution and Cancer, The Institute of Cancer Research, London, UK

**Keywords:** Acute lymphocytic leukaemia, Acute lymphocytic leukaemia

## Why prevention?

Despite advances in treatment efficacy, childhood cancers continue to exert a heavy toll in morbidity and mortality. Exploring new therapeutic options has been restrained by the relative rarity of these cancers coupled with their subgroup diversity and some reluctance to invest in drug development for rare cancers.

In adult cancers, later stage or metastatic disease remains largely intransigent with emergent drug resistance as the portal for malignant escape. Whilst novel combinatorial strategies involving immunotherapy, evolutionary or adaptive control might well thwart resistance [1–3], much emphasis is placed on early diagnosis and intervention where prospects for eradication or cure are more tangible.

But it has also been argued that since the belligerence of cancer is the result of a progressive evolutionary process with highly variable dynamics Plan A for cancer control should be prevention [4]. Or, to stop it before it gets started. In theory, this makes sense but for this to be plausible, let alone practicable, requires that we can identify critical components of the causal pathway that are amenable to interception. For many common adult cancers, including breast, prostate and colorectal this remains challenging. However, the consistent, causal links between smoking and lung cancer, skin cancer and UVB and cervical cancer and HPV [5] provide hugely encouraging examples of reduction in disease burden via education, prudent avoidance and, in the case of HPV, prophylactic vaccination. There is little doubt that cancer prevention is possible and can have a substantial, global impact on public health.

For paediatric cancers including both solid tumours and leukaemia, the picture has been different. Identifying causal pathways is extremely difficult for cancers that are both rare in prevalence and biologically diverse. Moreover, the common view that many if not most childhood cancers arise via stochastic, developmental errors compounded by inherited susceptibility [6] further dampens any enthusiasm to consider prevention as a possibility.

There is however one exception to this generally pessimistic perspective and that is with childhood acute lymphoblastic leukaemia (ALL). This is the most common type of paediatric cancer (around one-third of all cases) but is itself heterogeneous, originating from multi-lineage or lymphoid progenitors. Discriminating between these subtypes has been a key component of unravelling likely causal pathways. And for the most frequent subtype, B cell precursor ALL (~75% of total), a combination of basic biological investigations and large collaborative case/control epidemiological studies has delivered a plausible causal mechanism which illuminates prospects for prevention [7].

But first, a caveat. ALL has provided one of the real success stories in oncology. Universally lethal in the absence of effective treatment [8], this cancer has been transformed by stepwise, incremental gains via systematic clinical trials of combination chemotherapy with a current cure rate of around 90% [9]. So, why should we be interested in prevention? One glib sounding but the valid answer would be to say, ‘ask any parent of a patient’. The reality is that the treatment is traumatic for very young patients and their families, and toxic with some cost or deleterious trade off in terms of morbidity and long-term health impacts [10, 11]. The excellent prospects for curative treatment are massively important to the affected families but prevention, if possible, would surely be even better?

## Dissecting multifactorial causal mechanisms: where is the leverage?

**Fig. 1:**
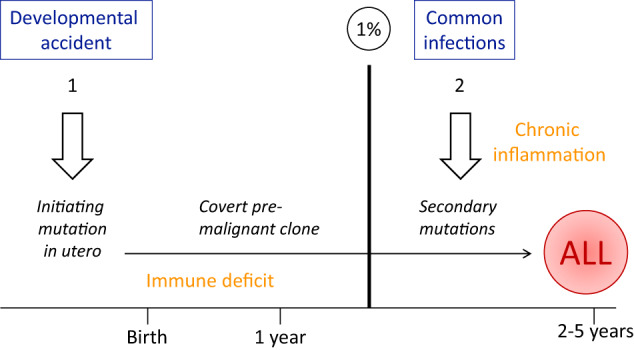
The two-hit model for B cell precursor ALL. Initiating genetic lesions are primarily ETV6-RUNX1 or hyperdiploidy, probably occurring as developmental accidents. They arise in utero possibly in foetal liver early B lineage lymphopoiesis [78]. Secondary mutations are primarily RAG-mediated copy number alterations. ~1% figure: ALL is initiated in utero at a rate that exceeds by 100-fold, the incidence of disease indicating a low penetrance and a critical role for factors promoting chronic inflammation and the secondary mutations. Adapted from [7]. See text for references.

Infection has, for almost a century, been considered a possible causal agent for childhood ALL [12]. But unlike leukaemias in cats, chickens and cattle [13], no specific transforming virus has been identified. Instead, the current model embodies a paradox, namely that although common infections may trigger or promote this cancer the key risk factor is actually a deficit of microbial exposure, in infancy and especially in more developed or affluent societies [7, 14]. This causal model of ALL is grounded in the evolutionary, natural history of the disease, considerations of how the immune system has evolved to respond to microbial exposures and extensive epidemiological assessment of risk variables that are surrogates for common microbial exposures. The detailed evidence has been summarised recently [7]. Figure 1 presents a pictorial version of the model.

Much of the historical approach to understanding why we get cancer has courted the implicit concept of singularity of cause. This makes no more sense than singularity of cure or a magic bullet. Most if not all cancers are likely to have a multifactorial causation involving exogenous or endogenous exposures, background genetics and chance, which sit alongside evolutionary contingencies or liabilities underpinning vulnerability [4]. And ALL is no exception. For a child to develop ALL the following factors may have to come into play, collectively.

1. The acquisition, in utero, of an initiating mutation, most commonly chromosomal hyperdiploidy or *ETV6-RUNX1* gene fusion [7]. The founder event is far more common (~100 times) than overt ALL [15, 16] and generates a persistent, covert and non-pathological pre leukaemic clone that can persist for up to at least 14 years [17]. The cause(s) of the initiating mutations is unknown but is suggested to be endogenous oxidative stress [7, 18].
2. A small fraction (~1%) of pre leukaemias initiated in utero progress to clinical ALL, usually between the ages of two and six, after they acquire additional mutations. The latter most commonly being recombinase enzyme (RAG 1, 2) driven copy number losses of genes involved in B lineage differentiation or cell cycle control [19, 20]. The model posits that these necessary secondary mutations are an indirect consequence of a dysregulated immune response or chronic inflammation consequent to common infections [7]. There is some mechanistic insight into how this might happen [21, 22]. The infections involved are not identified, though respiratory viruses have been implicated [23, 24]. Nursery groups and schools are a likely venue or hot spot for these infections. When all schools in Hong Kong were closed for a year due to the SARS pandemic in 2003 rates of ALL declined (but not brain tumour) as did notifiable common infections in children [25]. Widespread social restrictions during the 2020 COVID-19 pandemic might be expected to have a similar impact and is currently being assessed [26].
3. The abnormal immune response to infection in children that triggers progression to overt, clinical ALL is considered to be contingent upon a lack of microbial exposure in the first year of life which is required to prime the naïve immune network for well-regulated or balanced responses [7]. This scenario was first predicted based on immunological principles but then assessed and endorsed by case/control epidemiological studies and meta-analyses of the accumulated data [7, 27–29].

Risk is further modified by a number of inherited alleles expressed in blood cells [7, 30, 31] and may impact primarily by interacting epistatically with the endogenous mutations to drive transformation [7]. Dietary factors may also modify risk [32]. All of these variables are imbued with an element of chance and compound to provide a risk for ALL of around one in 2000 for the first 15 years of life.

## The microbiome link

**Fig. 2:**
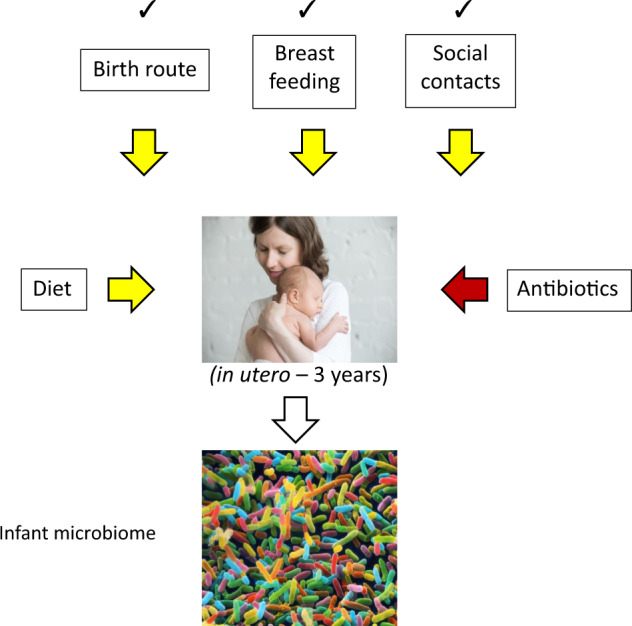
Environmental, life exposures that source and impact on the infant microbiome. Of the five critical factors, three (✓ in figure) have been implicated as risk factors in B cell precursor ALL. Birth route—vaginal versus caesarean. Note: diet and antibiotics also impact on the composition of the microbiome but those two variables have not been systematically evaluated for impact on the risk of ALL.

Of this list of risk variables, only one would seem to be potentially modifiable. This is the apparent deficit of microbial exposure in infancy. It has been unclear what these microbial infections might be and there is no association between documented pathological infections in infancy and risk of ALL. The surrogate, epidemiological variables for this exposure [7] have been day-care attendance (protective), protracted breastfeeding (protective), C section birth (increased risk) and in some but not all studies, birth order (higher risk for first borne). These variables each reflect a route via which babies and infants acquire their gut microbiomes (Fig. 2) [33–35], suggesting that the key deficit or risk factor for ALL in early life may reside in the acquisition and composition of the commensal gut microbes.

There is now substantial evidence that establishment of the gut microbiome at birth and over the first few years of life [34, 35] has profound and long-lasting effects on both metabolism and immune system function [36, 37]. The mechanisms involved are still being investigated but involve both metabolic products of bacteria [38] and direct microbial binding to Toll receptors on innate immune cells [39]. The key downstream consequence appears to be the activation of regulatory T cells that orchestrate the dynamics of immune responses [40, 41]. And the crucial, long-lasting impact is that the immune system’s complex network is ‘primed’ or hardwired for balanced responses and avoidance of chronic inflammation.

## ALL and other childhood diseases of ‘affluence’

Microbial dependency of the immune system, registered very early in life, is likely to be evolutionarily ancient [42] but several aspects of modern life styles in westernised countries, including childbirth and breastfeeding practices, antibiotic use, diet and social contacts have disrupted this arrangement resulting in less diverse microbiomes or dysbiosis [43, 44]. This evolutionary mismatch might be expected to have many important pathological consequences in both children and adults for both metabolism and immune function, one of which now seems likely to be childhood leukaemia. And for children with an immune priming deficit the risks or consequences are not just for ALL.

Childhood allergies and type 1 diabetes are both linked epidemiologically with a deficiency of early life microbial exposure [45, 46] and more recent studies have provided some direct evidence for dysbiosis of the gut microbiome in these conditions [47, 48]. The aetiological model for these childhood diseases was originally named the ‘hygiene hypothesis’ but it has become clear that risk is less to do with hygiene as ‘cleanliness’ and more to do with ‘mixed blessing’ lifestyle changes that diminish opportunities for exposure to both deleterious pathogens and beneficial commensals as ‘old friends’ [49, 50]. Microbiome dysbiosis can therefore be considered as an unintended and deeply paradoxical consequence of ‘progress’.

Childhood allergies and type 1 diabetes share many of the same early life risk factors as ALL mirroring routes of gut microbiome acquisition (Table 1). Incidence rates of all three childhood illnesses have increased over recent decades in developed societies and internationally track together with markers of affluence. Scandinavian countries topping the list [7, 45].

**Table 1:** Shared risk factors between ALL, type 1 diabetes and allergies in children.

Risk factor for ALL	Risk	Type 1 diabetes (ref.)	Allergy (ref.)
Day-care attendance	Down	+[72]^a^	+[73]
Breastfeeding	Down	+[74]^a^	+[75]^a^
C-section birth	Up	+[76]^a^	+[77]

Risk factors for ALL (reviewed in [7]) also reported (+) for type I diabetes or allergies. There are some caveats to this summary. There is some heterogeneity of results reported and variation in parameters measured that could be important for immune priming in infancy, e.g. age and time spent in day care and length of time breastfeeding. Type of allergies or asthma measured is another variable. These data merit further scrutiny.

^a^Meta-analysis study.

Could childhood ALL, allergies and type 1 diabetes, and possibly some other autoimmune diseases, such as multiple sclerosis (MS) [51], all share the same underlying, predisposing condition—an early life gut microbiome dysbiosis resulting in an immune priming deficiency? This concept might appear to be contradicted by the observation that they tend not to co-occur in families and have very distinct pathologies. But this could be explained by the impact of differing inherited susceptibility alleles plus distinctive triggering factors targeting separate tissues. The notion that all three childhood illnesses might share the same underlying fault could, if correct, have major implications, not least for therapeutic or preventative intervention.

## Prospects for microbiome boosting

Gut microbiome dysbiosis has also been linked in recent years to a number of common adult conditions including inflammatory bowel disease (IBD) and obesity [52]. Dysbiosis could also be involved in the considerable fraction of adult cancers associated with chronic inflammation [53, 54] with important implications for unpicking causation and treatment or prevention.

Notwithstanding the need for more exploration of the considerable complexities of the gut microbiome ecosystem, there is already clinical exploration of the potential clinical benefits of gut microbiome reconstitution or boosting. Examples include adult IBD [55], compensation for the microbiome deficit of C-section birth [56] and to combat the emergence of antibiotic resistant bacteria [57]. Gut microbiome reconstitution has been used for leukaemia patients having received bone marrow transplants coupled with microbiome crippling antibiotics [58]. Boosting of the gut microbiome may enhance the efficacy of immunotherapy in cancer [59].

Some of these clinical trials, and animal modelling, involve transfer of total stool samples or faecal microbiota transplants. This tactic may capture the microbial diversity of the gut microbiome, but standardised use and regulatory approval will require well defined, and non-pathogenic bacterial species. In this respect, it is encouraging that ‘keystone’ [60] bacterial species of the healthy infant microbiome ecosystem—*Bifidobacteria* sp., as well as *Lactobacilli*, administered with or without milk oligosaccharides, as synbiotic regimes, have provided clinical benefit or risk reduction to infants in the context of sepsis [61], allergies [62] and pre-term birth consequences [56, 63].

Collectively, these data raise the possibility that gut microbiome boosting might present a viable strategy for risk reduction or prevention in childhood ALL. A similar argument has been made for prophylactic intervention for type 1 diabetes in children [64]. But for this to be translated into practice requires additional questions to be addressed.

## The challenges ahead

First, there is a need for more direct evidence that the microbiome in patients developing ALL is indeed deficient or lacking in diversity. Prospective monitoring of a very large (tens of thousands) cohort of infants might provide that evidence and such studies are initiated or in planning phases to screen for multiple heath impacts. One study [65] reports that at diagnosis, patients with ALL do have a less diverse oral microbiome than controls. This study requires scale up, confirmation for the gut microbiome and also needs to accommodate the potentially confounding effects of prior antibiotic use or the disease process itself. A proof of principle demonstration that microbiome boosting can indeed prevent infection promoted ALL in an animal model that faithfully mimics the clinical disease in children would also be very encouraging. These models are currently under development [66] (MG, VC and AF unpublished). In this respect studies on rodent models of type 1 diabetes are more advanced than leukaemia with accumulating evidence for risk reduction via microbiome-based immune modulation [45, 67–71].

But even if these issues were resolved there are several other impediments to translation of this idea into a public health measure. First, there is the substantive issue of defining the precise bacterial mix or cocktail that might be effective, coupled with the challenge of delivery and safety. But, with common adult diseases primarily in mind this is now high on the agenda in both biotech industry and academia and is likely to be resolved soon. Second, there is the question of who would receive any potential protective treatment in infancy. Although the main risk factors for ALL are now recognised, it remains very difficult to identify individuals at risk in the population. Any prophylactic intervention might therefore have to be unselective or population wide. How would this be justified for a cancer that is rare and largely curable? It could be argued that the current suite of vaccines given to young children provide a precedent but this might prove unpersuasive.

There is one strategy that could be taken to both address this challenge and maximise potential benefit. This is to ask the audacious question of whether population wide microbiome boosting in infancy, with a single defined, and safe, bacterial preparation, might not deliver multiple health benefits, including risk reduction for childhood leukaemia, allergies and autoimmune disease. And, very likely, long-term benefits for adult health.
